# Influence of Genetic Polymorphisms on Clinical Outcomes of Glatiramer Acetate in Multiple Sclerosis Patients

**DOI:** 10.3390/jpm11101032

**Published:** 2021-10-15

**Authors:** María José Zarzuelo-Romero, Cristina Pérez-Ramírez, Yasmín Cura, María Isabel Carrasco-Campos, Luciana María Marangoni-Iglecias, María Carmen Ramírez-Tortosa, Alberto Jiménez-Morales

**Affiliations:** 1 Department of Pharmacy and Pharmaceutical Technology, Faculty of Pharmacy, University of Granada, 18001 Granada, Spain; mjzarzuelo@ugr.es; 2 Center of Biomedical Research, Department of Biochemistry and Molecular Biology II, Institute of Nutrition and Food Technology “José Mataix”, University of Granada, Avda. del Conocimiento s/n., 18016 Armilla, Granada, Spain; mramirez@ugr.es; 3 Pharmacogenetics Unit, Pharmacy Service, Virgen de las Nieves University Hospital, 18012 Granada, Spain; yacura@uc.cl (Y.C.); mariaisabelcarrascocampos@gmail.com (M.I.C.-C.); luciana_iglecias@hotmail.com (L.M.M.-I.); alberto.jimenez.morales.sspa@juntadeandalucia.es (A.J.-M.)

**Keywords:** multiple sclerosis, glatiramer acetate, myelin basic protein, response, treatment, polymorphisms, pharmacogenetics, medicine personalized

## Abstract

Multiple sclerosis (MS) is a chronic, inflammatory, demyelinating disease of autoimmune origin, in which inflammation and demyelination lead to neurodegeneration and progressive disability. Treatment is aimed at slowing down the course of the disease and mitigating its symptoms. One of the first-line treatments used in patients with MS is glatiramer acetate (GA). However, in clinical practice, a response rate of between 30% and 55% is observed. This variability in the effectiveness of the medication may be influenced by genetic factors such as polymorphisms in the genes involved in the pathogenesis of MS. Therefore, this review assesses the impact of genetic variants on the response to GA therapy in patients diagnosed with MS. The results suggest that a relationship exists between the effectiveness of the treatment with GA and the presence of polymorphisms in the following genes: *CD86*, *CLEC16A*, *CTSS*, *EOMES*, *MBP*, *FAS*, *TRBC1*, *IL1R1*, *IL12RB2*, *IL22RA2*, *PTPRT*, *PVT1*, *ALOX5AP*, *MAGI2*, *ZAK*, *RFPL3*, *UVRAG*, *SLC1A4*, and HLA-DRB1*1501. Consequently, the identification of polymorphisms in these genes can be used in the future as a predictive marker of the response to GA treatment in patients diagnosed with MS. Nevertheless, there is a lack of evidence for this and more validation studies need to be conducted to apply this information to clinical practice.

## 1. Introduction

Multiple sclerosis (MS) is a chronic, inflammatory, immune-mediated disease that causes the demyelination and neurodegeneration of the axons of central nervous system (CNS) neurons. This leads to exacerbations and remissions and/or the insidious progression of neurological symptoms, causing disability and impairing the patient’s quality of life [1,2,3,4]. Its origin is unknown, but it is believed that CD4^+^ and CD8^+^ T-cell infiltration, B cells, macrophages and the disturbance of redox homeostasis are implicated in the development and progression of the disease [5,6,7,8]. Its diagnosis is based on medical history and neurological examination but, due to the heterogeneity of MS symptoms and the variable outcomes, an accurate diagnosis is currently challenging [9,10]. Numerous cerebrospinal fluid biomarkers are proposed for the diagnosis and prognosis of the disease, such as oligoclonal bands, oxidative enzymes and the IgG index, but only a few are clinically implemented [6,7,8,11,12].

The disease is one of the main causes of disability in young people and is most prevalent between the ages of 20 and 50 and in women [13]; it affects more than 2 million people worldwide [13,14].

Multiple sclerosis can be classified into different types according to the progression of the disease: relapsing-remitting multiple sclerosis (RRMS), primary progressive multiple sclerosis (PPMS) and secondary progressive multiple sclerosis (SPMS) [15,16]. Approximately 80% of the patients diagnosed with MS present with RRMS, characterized by unpredictable and recurrent acute or subacute episodes of neurological disturbances that can last for days or weeks, alternated with stable phases which can even show slight improvements [17,18]. Most patients with RRMS will eventually develop SPMS, with clearly defined exacerbations or flare-ups in which the disease becomes considerably more severe and follows a constant linear progression. Between 15% and 20% of patients have the PPMS type, in which there are no clear exacerbations and the progression is slow and gradual from the beginning, with no phases in which the patient experiences an improvement of their disease [17,18].

Although there is currently no cure for multiple sclerosis, there are treatments that slow down the course of the disease and mitigate its symptoms. They can be divided into classical therapies, such as glatiramer acetate (GA) and interferon beta, and new therapies [19,20,21,22]. GA is one of the most commonly used medications in first-line treatment. This is a synthetic copolymer composed of random sequences created by the polymerization of four L-amino acids: glutamic acid, alanine, lysine, and tyrosine, forming a structure similar to that of the myelin basic protein (MBP) [17,18]. This drug represents the first non-steroidal immunomodulator, other than interferon, that modulates the immune system process underlying the pathogenesis of MS. Although its precise mechanism of action is not known, its effects seem to be mediated by the production of antibodies.

**Figure 1:**
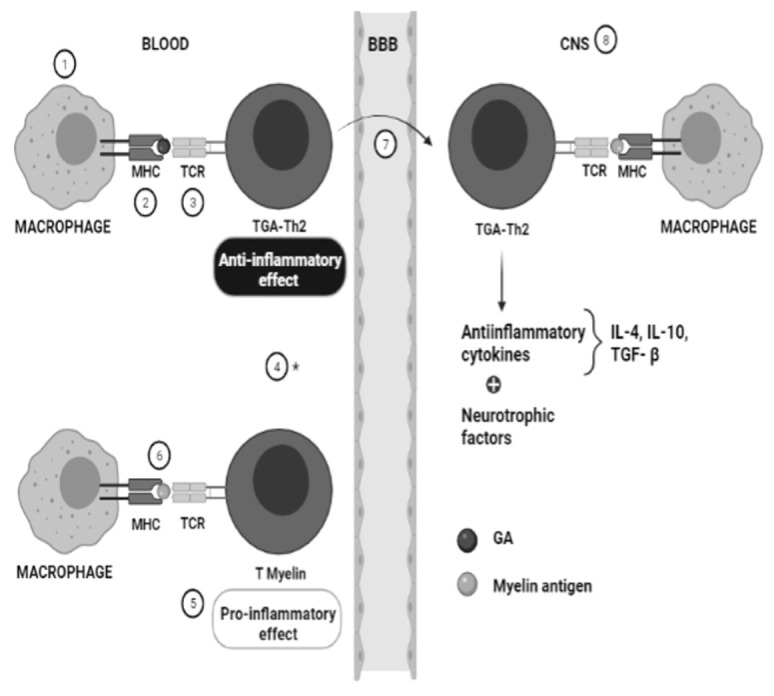
Mechanism of action of GA in treatment of multiple sclerosis. GA modulates the immune response and alters T-cell differentiation by competing with MBP in the major histocompatibility complex responsible for antigen presentation. Numbers represent the action site of genes with probable potential involvement in the pharmacokinetics and pharmacodynamics of GA therapy, patho-etiology of MS, apoptosis and neuroprotection, and CNS repair. 1. APCs: CD86, CCR5, CLEC16A; 2. MHC: HLA-DRB1, CTSS; 3. TCR: TRBC1; 4. T-cell regulation: EOMES, PVT1, CCR5, PTPRT, UVRAG; 5. Pro-inflammatory pathway: ALOX5AP, IL12RB2, IL22RA2, IL1RL1; 6. MPB: MPB, CTSS; 7. T-cell migration: CCR5; 8. CNS: MAGI2, RFLP3, SLAC1A4; * Other: Apoptosis mediatiors: CD95, ZAK. APC: antigen-presenting cells; BBB: blood–brain barrier; CNS: central nervous system.

The mechanism of action of GA is not fully clarified; however, it is believed that it has a dual mechanism of action reducing inflammation in the periphery and the CNS and causing the release of neurotrophic factors within the CNS. In the periphery, GA competes with myelin antigens in APC cells (dendritic cells, macrophages and b cells) for binding at the major histocompatibility complex (MHC), which hinders the proliferation of myelin-reactive T cells and their capacity to secrete proinflammatory cytokines, while activating GA-induced T cells, mediating the anti-inflammatory protective pathways which suppress the disease. Numerous studies suggest that the main mechanism of GA is its ability to change the response of T cells from the pro-inflammatory to the anti-inflammatory pathway (also called Th1 to Th2 shift). GA-induced immune cells cross and accumulate in the CNS where they secrete anti-inflammatory cytokines (IL-4, IL-10, TGF-β) in response to myelin antigens [23,24,25,26]. (Figure 1). According to the European Committee for Treatment and Research in Multiple Sclerosis, GA is recommended as a first-line treatment in patients with clinically isolated syndrome (CIS) and abnormal magnetic resonance imaging with lesions suggestive of MS [27]. GA significantly reduces the number of relapses in patients with RRMS and is also an efficacious and safe treatment [28,29,30]. However, the response rate is around 30–55% [31,32,33,34]. Consequently, the genetic alterations in the genes involved in the pathological environment of the disease, or the pharmacodynamics, metabolism, or mechanism of action of GA, may influence the effectiveness of this drug [18,26].

The following section contains a review of the pharmacogenetic studies of the impact of genetic variants on the response to treatment, with GA in patients diagnosed with MS (Table 1).

## 2. Materials and Methods

A PubMed search included the key words: “multiple sclerosis”, “glatiramer acetate”, together with “polymorphisms” and “response”. Data regarding the gene, year of publication, number of patients, population, polymorphism, drugs, overall response rate (odds ratio, 95% confidence interval and *p*-value), and the genotype associated with the response were recorded.

## 3. Pharmacogenetics of Glatiramer Acetate in MS

**Figure 2:**
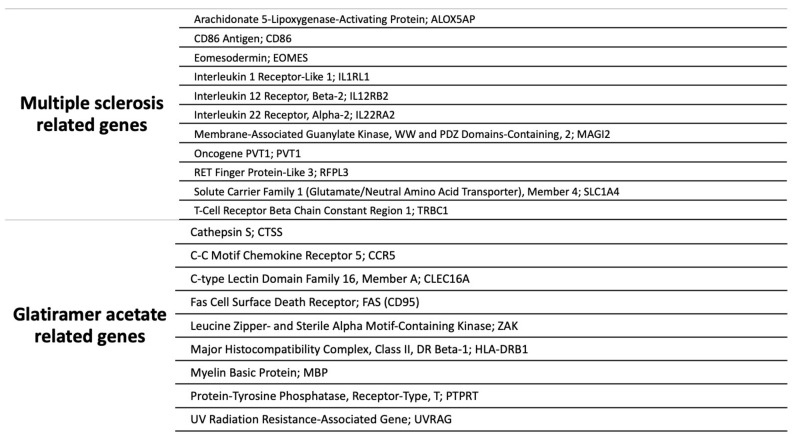
Genes involved in pharmacogenetics of AG.

Pharmacogenetics is the study of how genetic variants influence drug response [35,36,37,38]. The use of GA as one of the first-line treatments for MS has significantly improved outcomes but has also shown great variability in response among patients [39,40,41,42]. This variability may be due to the presence of genetic alterations in the candidate genes involved in: (a) the GA mechanism of action, (b) MS pathogenesis. Numerous studies reflected the notion that genetic alterations may be responsible for these inter-individual differences in response [18,26,43,44,45]. Consequently, various research studies conducted in recent years evaluated the associations between variants of the *ALOX5AP*, *CTSS*, *CCR5*, *CD86*, *CLEC16A*, *EOMES*, *FAS*, *IL1RL1*, *IL12RB2*, *IL22RA2*, *ZAK*, *HLA-DRB1*, *MAGI2*, *MBP*, *PVT1*, *PTPRT*, *RFPL3*, *SLC1A4*, *TRBC1* and *UVRAG* genes and the responses to treatment with GA (Figure 1 and Figure 2) (Table 1).

### 3.1. Multiple Sclerosis Related Genes

#### 3.1.1. Arachidonate 5-Lipoxygenase-Activating Protein; ALOX5AP

The arachidonate 5-lipoxygenase-activating protein gene (*ALOX5AP*) is located in the 13q12.3 region of the chromosome [46]. This gene codes for a 5-lipoxigenase activating protein and is thus involved in the synthesis of leukotrienes, implicated in a variety of inflammatory responses, including asthma, arthritis, and psoriasis. A recent study of the effect of inflammatory mediators belonging to the lipoxygenase family on the pathogenesis of MS showed higher levels of 15-lipoxygenase in patients with MS [47]. Furthermore, a study showed that GA may induce inflammation and immediate hypersensitivity through ALOX5AP [48].

A study conducted by Ross et al. in 2017 analyzed several polymorphisms in 1171 Caucasian patients diagnosed with RRMS and treated with GA (from Argentina, Belgium, Bulgaria, Canada, Croatia, the Czech Republic, Estonia, Finland, France, Georgia, Germany, Hungary, Israel, Italy, Latvia, Lithuania, The Netherlands, Poland, Romania, Russia, South Africa, Spain, Ukraine, the UK, and the United States); 639 patients in the Glatiramer Acetate Low-frequency Administration (GALA) group, to whom 40 mg/mL GA was administered for twelve months and compared three times per week with a placebo; and 532 patients in the FORTy mg Efficacy of glatiramer acetate (FORTE) group, where the administration of 20 mg/mL GA was compared with 40 mg/mL for 12 months. The rs10162089 (T > C) polymorphism showed a strong association with the response to GA in the GALA study. Similarly, this rs10162089 (T > C; intron variant) polymorphism showed a significant association with treatment in the FORTE study (Table 1) [49].

#### 3.1.2. CD86 Antigen; CD86

The CD86 antigen gene (*CD86*) is located on chromosome 3q13.33 [50]. The CD86 molecule is a glycoprotein which acts as a receptor on the membrane of antigen-presenting cells (APCs). It provides the costimulatory signals needed for T-cell activation [28,51]. An increase in CD86 was observed in the blood cells of patients with MS, inducing the proinflammatory response [52]. Interestingly, a study reported that GA suppressed dendritic cell maturation through the upregulation of HLA-DR and CD86 [53].

A study involving 35 Caucasian patients with RRMS (from Belgium, Canada, The Netherlands, Italy, and the UK) found that the C allele of the rs1129055 (T > C; missense variant) polymorphism was associated with a worse response to GA treatment [17]. In addition, the T allele of the rs2001791 (T > C; intron variant) polymorphism was associated with a greater GA response in a study with 48 Caucasian patients (from Belgium, Canada, The Netherlands, Italy, and the UK) (Table 1) [17].

#### 3.1.3. *Eomesodermin*; *EOMES*

*The eomesodermin gene (EOMES)* is located in the 3p24.1 region [54]. This gene is a member of the TBR1 (T-box, brain, 1) subfamily of T-box genes that plays a central role during cortical neurogenesis. *EOMES* encodes the transcription factor Eomesodermin (Eomes) and is predominantly expressed in NK cells and T cells. Though its exact function is not yet clarified, it was shown to be implicated in the differentiation, function, and homeostasis of effector immune cells. On the one hand, the reduced expression of *EOMES* was shown to be significantly associated with MS [55]. On the other hand, it was observed that Th with a high expression of Eomes was significantly increased in the peripheral blood of SPMS patients. These cells were also found to infiltrate the brain tissues in SMPS autopsy samples, suggesting a pathogenic role in SPMS development and progression [56].

In a Russian population of 296 RRMS patients treated with GA for 2 or more years, the rs2371108 (G > T; intergenic variant) T allele was associated with event-free status, which was the optimal clinical response in MS patients (Table 1) [57].

#### 3.1.4. Interleukin 1 Receptor-like 1; IL1RL1

The interleukin 1 receptor-like 1 gene (*IL1RL1*) is located in the 2q12.1 region [58]. IL1RL1 is a receptor belonging to the IL1 family which is selectively expressed in Th2 cells and mast cells. It mediates the biological effects of IL33, a member of the IL1 family, which leads to the production of Th2-associated cytokines [59]. IL1RL1 plays an important role in autoimmune and inflammatory diseases, such as MS [60]. A study reported that GA induced the production of IL1R antagonists in monocytes [61].

A study of 48 Caucasian patients with RRMS (from Belgium, Canada, The Netherlands, Italy, and the UK) evaluated the influence of the rs956730 (A > G; intron variant) polymorphism on the response to GA treatment, showing a significant response in patients carrying the A allele (Table 1) [17].

#### 3.1.5. Interleukin 12 Receptor, Beta-2; IL12RB2

The interleukin 12 receptor, beta-2 gene (*IL12RB2*) is located in the 1p31.3 region of the chromosome [62]. The protein encoded by this gene belongs to the Interleukin-12 (IL-12) complex. The subunit, *IL12RB1,* is constitutively expressed in both Th1 and Th2 lymphocytes. However, *IL12RB2* plays a different role in the differentiation of Th1 cells, being more strongly expressed in these cells, and can be induced by the activation of the antigen receptors or by IL-12 and alpha interferon [63]. Therefore, the expression of *IL12RB2* is implicated in immune-mediated diseases such as MS [64] and it is reported that GA reduces IL-12 production [53].

A study in 34 Caucasian patients (from the United States of America) with the G allele of the rs946685 (G > A; intron variant) polymorphism, found a significant association with GA treatment response (Table 1) [17].

#### 3.1.6. Interleukin 22 Receptor, Alpha-2; IL22RA2

The *interleukin 22 receptor, alpha-2 gene (IL22RA2)* is located on chromosome 6q23.3 [65]. *IL22RA2* encodes a soluble class II cytokine receptor and naturally occurring IL22 antagonist [65]. IL22RA2 is associated with the neuroinflammation and effects on MS susceptibility and severity [66]. A study of 296 RRMS patients (from Russia) with the GG genotype of the rs202573 (G > A; intron variant) polymorphism, treated with GA, found a significant association with event-free status (Table 1) [57].

#### 3.1.7. Membrane-Associated Guanylate Kinase, WW and PDZ Domains-Containing, 2; MAGI2

The membrane-associated guanylate kinase, WW and PDZ domains-containing 2 gene (*MAGI2*) is located in the 7q21.11 region of the chromosome [67]. The *MAGI2* gene serves as a scaffold for the assembly of the neurotransmitter receptors and cell adhesion proteins [68]. In addition, it seems to play a part in regulating the signals in neuronal cells, and thus seems to be involved in MS [69].

In a study (GALA) of 639 patients with RRMS an association was observed between the rs16886004 (A > G; intron variant) polymorphism, as well as a strong significant response to GA treatment. In the FORTE study, a significant association with the treatment was also found for the same polymorphism in 532 Caucasian patients (from Argentina, Belgium, Bulgaria, Canada, Croatia, the Czech Republic, Estonia, Finland, France, Georgia, Germany, Hungary, Israel, Italy, Latvia, Lithuania, The Netherlands, Poland, Romania, Russia, South Africa, Spain, Ukraine, the UK, and the United States) (Table 1) [49].

#### 3.1.8. Oncogene PVT1; PVT1

The plasmacytoma variant translocation oncogene (*PVT1*) is located in the 8q24.21 region, which is a preferred site for chromosomal rearrangements in cancers and incorporates multiple risk loci for MS [70,71]. The lymphocyte activation through the involvement of PVT1 is implicated in adaptive immunity and has implications for autoimmune diseases (MS, inflammatory bowel disease, rheumatoid arthritis) [72,73]. In a study involving 296 RRMS patients (from Russia) treated with GA, the A allele of the rs2114358 (A > G; intron variant) polymorphism was associated with event-free status (Table 1) [57].

#### 3.1.9. RET Finger Protein-like 3; RFPL3

The RET finger protein-like 3 gene (*RFPL3*) is located in the region 22q12.3 [74]. The human *RFPL1, RFPL2*, and *RFPL3* genes are highly expressed in neurogenesis during the neural differentiation of embryonic stem cells and contribute to changes in the organization and/or size of the neocortex [74].

The effect of the rs73166319 (C > T; regulatory region variant) polymorphism on the response to GA was evaluated in 1171 patients with RRMS (from Argentina, Belgium, Bulgaria, Canada, Croatia, Czech Republic, Estonia, Finland, France, Georgia, Germany, Hungary, Israel, Italy, Latvia, Lithuania, Netherlands, Poland, Romania, Russia, South Africa, Spain, Ukraine, UK, and United States) and a significant association was found (Table 1) [49].

#### 3.1.10. Solute Carrier Family 1 (Glutamate/Neutral Amino Acid Transporter), Member 4; SLC1A4

The solute carrier family 1 member 4 gene (*SLC1A4*), located in the 2p14 region of the chromosome, encodes the SLC1A4 Na(+)-dependent amino acid transporter, which transports four L-amino acids: serine, alanine, cysteine, and threonine. In the brain, L-serine is synthesized by astrocytes and shuttled into the neuronal cells by the SLC1A4 transporter [75]. Demyelination, involved in MS, could be due to the inflammatory response related to expression of SLC1A4 [76].

The rs759458 (G > A; missense variant) polymorphism shows an association with GA response in two studies involving 532 and 639 Caucasian patients with RRMS (from Argentina, Belgium, Bulgaria, Canada, Croatia, the Czech Republic, Estonia, Finland, France, Georgia, Germany, Hungary, Israel, Italy, Latvia, Lithuania, The Netherlands, Poland, Romania, Russia, South Africa, Spain, Ukraine, the UK, and the United States) (Table 1) [49].

#### 3.1.11. T-Cell Receptor Beta Chain Constant Region 1; TRBC1

The T-cell receptor is a heterodimeric glycoprotein comprising two pairs of polypeptide chains (α and β) present on the cell surface of T lymphocytes, responsible for the T-cell recognition of antigens presented by MHC in the APC cells. Because of their essential role as members of the “tri-molecular complex”, formed by HLA-DRB1, MPB and T-cell receptors α and β, the genes coding the T-cell receptor α and β chains are identified as primary candidate genes for the prediction of the susceptibility to MS and GA therapeutic responses. Of both the polypeptide chain genes, only the T-cell receptor beta chain constant region 1 gene (*TRBC1*) polymorphisms are associated with MS susceptibility [77]. *TRBC1* is located on chromosome 7q34 [77]. Hockertz et al. found that the susceptibility to MS was associated with a gene linked to the variable region of TCRB [78]. Therefore, TRBC1 played an important role in the immune processes that controls the pathology of MS.

A study comprising 31 Caucasian patients with RRMS (from the United States of America) evaluated the association of the rs71878 (C > T; synonymous variant) polymorphism with the GA response, showing a greater response in patients carrying the C allele (Table 1). The association detected was proven to be GA-specific as it was only significant in the GA-treated group and not in the placebo-treated group [17].

### 3.2. Glatiramer Acetate Related Genes

#### 3.2.1. Cathepsin S; CTSS

The cathepsin S gene (*CTSS*), located in the 1q21.3 region, belongs to the lysosomal cysteine protease family [79]. The protein it encodes acts as an endoprotease, which breaks down the invariant chain of the MHC class II, both in dendritic cells and in the microphages, before they are presented to the antigen. In addition, CTSS has an important role in antigen processing, as it can modify the extracellular matrix in tissues when it is secreted [80]. Attributing the mechanism of action of GA to Th2 cells raises the hypothesis that genetic variations in *CTSS* may affect the response to GA treatment.

Grossman et al. carried out a pharmacogenetic study on 101 Caucasian patients diagnosed with RRMS (from Belgium, Canada, The Netherlands, Italy, and the UK) [10]. This study showed a strong significant association in 43 patients between the G allele of the rs2275235 (G > A; intron variant) polymorphism and GA response [17]. Furthermore, for the rs1415148 (A > G; intron variant) polymorphism a significant association was found in 47 patients between the A allele and the response to the drug (Table 1) [17].

#### 3.2.2. C-C Motif Chemokine Receptor 5; CCR5

The C-C motif chemokine receptor 5 gene (*CCR5*) is located on chromosome 3p21.31 [81]. CCR5 is a transmembrane protein encoded by the *CCR5* gene. This gene is mainly expressed in cells derived from bone marrow, such as T lymphocytes, macrophages, and dendritic cells [82]. *CCR5* plays a key role in activating Th1 lymphocytes at the inflammation sites [83], as occurs in MS lesions, where the expression of CCR5 increases [84]. GA may downregulate the expression of CCR5 receptors and block their migration [85].

There are no studies assessing the effect of genetic alterations in this gene on GA response. However, a study comprising 285 Caucasian patients with MS (from Russia) evaluated the effect of nine polymorphisms in genes that coded for important pro- and anti-inflammatory cytokines (*TNF*, *IFNG*, *TGFB1*, *IFNB1*), cytokine receptors (*IFNAR1*, *IL7RA*, *CCR5*), *CTLA4*, and *DRB1*. A higher risk of ineffectiveness in the response to GA treatment was shown by the allelic combination DRB1*15 + CCR5*d + TGFB1*T + IFNAR1*G and DRB1*15 + CCR5*d + TGFB1*T. The migration of GA-specific lymphocytes with an anti-inflammatory effect through the BBB into the inflammatory sites in the CNS, was controlled by CC chemokines encoded by CCR5. The CCR5*d variant was a 32 bp gene deletion (w → del32) that resulted in a frameshift and nonfunctional truncated protein. The lack of protein function could hinder GA-specific T-cell migration to their action sites in the SNC which may contribute to GA ineffectiveness. Although, CCR5*d alone did not show a significant association with GA response, a significant influence was observed as part of an allelic combination. This suggested the cooperative effect of these genetic variants in the penetration capacity of GA-specific T-cells [86].

#### 3.2.3. *C-Type Lectin Domain Family 16, Member A; CLEC16A*

The *C-type lectin domain family 16, member A* gene (*CLEC16A*) is located on chromosome 16p13.13 [87]. This gene is extensively expressed in various immune cells in certain parts of the brain. It is broadly expressed by antigen-presenting cells and participates in the HLA-II pathway in B cells [88,89]. The mapping analyses of MS-associated single-nucleotide polymorphisms (SNPs) in the 16p13 region point to *CLEC16A* SNPs as the most strongly MS-associated genetic variants in this gene-rich region, and CLEC16A levels are strongly elevated in multiple sclerosis patients [90]. A study reported that *CLEC16A* may regulate the antigen presentation required for the formation of GA-reactive immune cells [44]. In a Russian population of 296 RRMS patients undergoing GA treatment the A allele of the rs6498169 (G > A; intron variant) polymorphism was associated with event-free status (Table 1).

#### 3.2.4. Fas Cell Surface Death Receptor; FAS (CD95)

The Fas cell surface death receptor gene (*FAS*) is located in the 10q23.31 region of the chromosome [91]. The Fas antigen is expressed in T cells and induces cell death in an autonomous manner consistent with apoptosis. It was shown that the production of natural regulatory T cells could be impaired in MS, which would lead to the silencing of autoreactive T and B cells, and thus to the development of autoimmunity [92]. Moreover, these autoreactive T cells cross the blood–brain barrier could cause demyelination through a cascade of events mediated by Fas [93]. A study also revealed that GA played a crucial role in B-cells by downregulating Fas [94].

In a study involving 47 Caucasian patients with MS (from Belgium, Canada, The Netherlands, Italy, and the UK), the C allele of the rs982764 (T > C; intron variant) polymorphism was associated with a significant positive response to GA (Table 1) [17].

#### 3.2.5. Leucine Zipper- and Sterile Alpha Motif-Containing Kinase; ZAK

The leucine zipper- and sterile alpha motif-containing kinase gene (*ZAK*) is located on chromosome 2q31.1. ZAK is a serine/threonine-specific kinase belonging to the mitogen-activated protein triple kinase (MAP3K) family [95]. This serine/threonine kinase activates the c-Jun N-terminal kinase 1/stress-activated protein kinase (JNK/SAPK1) and the nuclear factor kappa light chain in β cells (NFĸβ) pathways [96]. The overexpression of *ZAK* gives rise to apoptosis and is associated with the cell division and cerebral lesions involved in MS [96]. Stress and inflammation are mechanisms of *ZAK* related to the mechanism of action of GA [97].

In the GALA pharmacogenetic study a significant association of the rs139890339 (C > T; intron variant) polymorphism with GA response was observed in 639 patients with RRMS [49]. Similarly, in the FORTE study a significant association with GA response for the same polymorphism was observed in 532 patients (Table 1) [49].

#### 3.2.6. Major Histocompatibility Complex, Class II, DR Beta-1; HLA-DRB1

The major histocompatibility complex, class II, DR beta-1 gene (*HLA-DRB1*) is located on chromosome 6p21.32 [98]. The major histocompatibility complex class II molecules are alpha/beta heterodimeric cell surface proteins whose function is to present the processed foreign antigens to T cells [99]. The inhibitory effects of GA on the induction of MHC class II are described [48]. It is suggested that HLA DRB1*1501 may favorably influence GA effects in the T cell receptor, triggering the polarization to Th2 cells [100].

The most significantly MS-associated SNP is rs3135391 (A > G; synonymous variant), a Class II SNP known to tag the HLA-DRB1*15:01 allele. In the studies conducted by Ross et al. they examined this polymorphism, and a relapse-free response was obtained both in the GALA study and in FORTE [49].

The rs3135388 (A > C, G, T; intergenic variant) polymorphism was also investigated in another study involving 332 American patients with RRMS undergoing GA treatment, and a greater therapeutic response was observed in patients with the AA genotype (Table 1) [100].

#### 3.2.7. Myelin Basic Protein; MBP

The myelin basic protein gene (*MBP*) is located on chromosome 18q23 [101]. MBP is the second most abundant protein in the CNS, responsible for the adhesion of the layers comprising the myelin that covers nerve tissues to maintain their structure [102]. In demyelinating diseases, such as MS, there are antibodies which attack MBP, causing the destruction of the protective myelin layer and leaving the nerves more exposed and unable to transmit signals to and from the brain [102]. GA is composed of polymers with a composition similar to that of MBP. These polymers perform immunomodulatory activities in MS by inhibiting the binding of MBP to the MHC and interrupting T-cell activation [101,103].

A study comprising 32 Caucasian patients with RRMS (from Belgium, Canada, The Netherlands, Italy, and the UK) assessed the association of the rs470929 (T > C; intron variant) polymorphism with GA response, finding a significant association in patients carrying the T allele [17]. The rs1789084 (T > C; intron variant) polymorphism also showed an association with GA response in two studies involving 639 and 532 Caucasian patients with RRMS (from Argentina, Belgium, Bulgaria, Canada, Croatia, the Czech Republic, Estonia, Finland, France, Georgia, Germany, Hungary, Israel, Italy, Latvia, Lithuania, The Netherlands, Poland, Romania, Russia, South Africa, Spain, Ukraine, the UK, and the United States) (Table 1) [49].

#### 3.2.8. Protein-Tyrosine Phosphatase, Receptor-Type, T; PTPRT

The protein-tyrosine phosphatase receptor-type T gene (*PTPRT*) is located in the 20q12-q13 region of the chromosome [104]. The protein encoded by this gene belongs to the protein tyrosine phosphatase (PTP) family. These enzymes act as signaling molecules which regulate a large variety of cellular processes, notably cell growth, differentiation, and the mitotic cycle, among others [105]. Therefore, PTPs perform a range of complex functions in the immune system, and the polymorphisms in the *PTPRT* gene can give rise to a modification of the response to GA.

The rs117602254 (C > T; intron variant) polymorphism showed an association with GA response in two studies of 532 and 639 Caucasian patients with RRMS (from Argentina, Belgium, Bulgaria, Canada, Croatia, the Czech Republic, Estonia, Finland, France, Georgia, Germany, Hungary, Israel, Italy, Latvia, Lithuania, The Netherlands, Poland, Romania, Russia, South Africa, Spain, Ukraine, the UK, and the United States) (Table 1) [49].

#### 3.2.9. UV Radiation Resistance-Associated Gene; UVRAG

The UV radiation resistance-associated gene (*UVRAG*) is located on chromosome 11q13.5 [106]. It codes for a protein associated with the resistance to ultraviolet (UV) radiation. The UVRAG protein was identified as a regulator of peripheral naïve T-cell homeostasis [107]. Therefore, the genetic alterations in the *UVRAG* gene may give rise to a modification of GA response [107].

A study found a statistically significant association of the rs80191572 (A > C, G; intron variant) polymorphism with the response to GA in 639 Caucasian patients diagnosed with RRMS (from Argentina, Belgium, Bulgaria, Canada, Croatia, the Czech Republic, Estonia, Finland, France, Georgia, Germany, Hungary, Israel, Italy, Latvia, Lithuania, The Netherlands, Poland, Romania, Russia, South Africa, Spain, Ukraine, the UK and the United States) [27]. This same polymorphism also showed a significant association with GA response in 532 Caucasian patients (Table 1) [49].

## 4. Conclusions

Multiple sclerosis (MS) is a chronic inflammatory demyelinating disease of autoimmune origin, in which inflammation and demyelination lead to neurodegeneration and progressive disability [1,2,3,4]. Currently there is no cure for multiple sclerosis, although there are treatments, such as GA, that slow down the course of the disease and mitigate its symptoms.

However, approximately 50% of patients with MS do not respond to GA treatment and there is a great variability in the responses between different patients. Both the etiopathogenesis of MS and the mechanism of action of GA are related to autoimmune processes, including inflammatory and oxidative stress processes. The present review focuses on the identification of genetic factors that may play a crucial role in the prediction of the response to GA.

Over the last years, most studies in MS focused on the identification of pharmacogenetics markers to predict the drug response of disease-modifying therapies, such as GA. Drug response is affected by pharmacokinetic, pharmacodynamic, pathological, and environmental factors; the drug mechanism of action, formulation, and route of administration; and patient characteristics. The genetic alterations in the genes involved in the pathological environment of the disease or the pharmacodynamics, metabolism, or mechanism of action of GA may influence the effectiveness of this drug and may be responsible for these inter-individual differences in response. Thus, identifying these genetic factors may play a crucial role in predicting the response to GA.

The association between gene polymorphisms and the variability in drug response was extensively investigated. Specifically, polymorphisms in *CD86* (rs1129055), *CLEC16A* (rs6498169), *CTSS* (rs2275235 and rs1415148), *EOMES* (rs2371108), *MBP* (rs470929 and rs1789084), *FAS* (rs982764), *TRBC1* (rs71878), *IL1R1* (rs956730), *IL12RB2* (rs946685), *IL22RA2* (rs202573), *PTPRT* (rs1117602254), *PVT1* (rs2114358), *ALOX5AP* (rs10162089), *MAGI2* (rs16886004), *ZAK* (rs139890339), *RFPL3* (rs73166319), *UVRAG* (rs80191572), *SLC1A4* (rs759458), and HLA-DRB1*1501 (rs3135391 and rs3135388) showed a statistically significant association with a response to GA treatment in patients with MS. Overall, we summarized the gene polymorphisms that were shown to play a significant role in the effectiveness of GA in MS patients.

The studies included in this review were selected in a systematic and unbiased manner. However, there were several limitations to this review. Firstly, many of the existing studies have a small sample size, which makes it difficult to accurately interpret the role of these genes in the response to GA treatment. Secondly, the studies show a lack of uniformity in the definition of treatment response and too short of a follow-up period for the results to be generalized. Finally, MS is a complex disease and the GA mechanism of action is not fully understood; consequently, gene polymorphisms may have a cumulative effect on the response to GA treatment and may need to be evaluated simultaneously to identify responders. Consequently, additional studies assessing multiple gene polymorphisms in RRMS patients with a uniform definition of response, longer-term follow-up, and larger sample sizes are needed to confirm these results.

In conclusion, the efforts to identify groups of relevant biomarkers in patients diagnosed with MS and treated with GA are beginning to show associations with response, such as the *CD86*, *CLEC16A*, *CTSS*, *EOMES*, *MBP*, *FAS*, *TRBC1*, *IL1R1*, *IL12RB2*, *IL22RA2*, *PTPRT*, *PVT1*, *ALOX5AP*, *MAGI2*, *ZAK*, *RFPL3*, *UVRAG*, *SLC1A4*, and HLA-DRB1*1501 gene polymorphisms that could be used in the future as predictive markers of response. However, there is a lack of evidence and more validation studies need to be conducted to transfer this information to clinical practice.

## Figures and Tables

**Table 1:** Influence of gene polymorphisms on glatiramer acetate therapy in Caucasian patients with multiple sclerosis.

Gene	Year	N	Ethnicity	Polymorphism	Overall Response Rate			PMID
					*p*-Value	OR (95% CI)	Genotype Associated	
*ALOX5AP*	2017	639	Multinational *	rs10162089	0.008	1.56	T	28569182
		532	Multinational *	rs10162089	0.032	1.58	T	
*CD86*	2007	35	Belgium, Canada, The Netherlands, Italy, and the UK	rs1129055	0.022	6.28 (1.3–30.3)	C	17622942
		48	Belgium, Canada, The Netherlands, Italy, and the UK	rs2001791	0.062	8.3 (0.9–77.0)	T	
*CLEC16A*	2017	296	Russian	rs6498169	0.025	2.38 (1.08–5.27)	A	29095108
*CTSS*	2007	43	Belgium, Canada, The Netherlands, Italy, and the UK	rs2275235	0.014	11.59 (1.6–81.9)	G	17622942
		47	Belgium, Canada, The Netherlands, Italy, and the UK	rs1415148	0.009	6.85 (1.6–29.2)	A	
*EOMES*	2017	296	Russian	rs2371108	0.018	2.00 (1.09–3.66)	T	29095108
*FAS*	2007	47	Belgium, Canada, The Netherlands, Italy, and the UK	rs982764	0.050	2.97 (1.0–8.8)	C	17622942
*IL1RL1*	2007	48	Belgium, Canada, The Netherlands, Italy, and the UK	rs956730	0.025	5.81 (1.2–27.1)	A	17622942
*IL12RB2*	2007	34	United States of America	rs946685	0.027	0.24 (0.07–0.85)	G	17622942
*IL22RA2*	2017	296	Russian	rs202573	0.008	2.08 (1.18–7.41)	GG	29095108
*HLA-DRB1*	2011	332	United States of America	rs3135388	0.015	2.7 (1.2–6.0)	AA	21115201
*MAGI2*	2017	639	Multinational *	rs16886004	0.002	2.15	A	28569182
		532	Multinational *	rs16886004	<0.001	5.56	A	
*MBP*	2007	32	Belgium, Canada, The Netherlands, Italy, and the UK	rs470929	0.040	5.3(1.1–25.9)	T	17622942
	2017	639	Multinational *	rs1789084	0.036	0.7	T	28569182
*PTPRT*	2017	639	Multinational *	rs1117602254	0.004	0.21	C	28569182
		532	Multinational *	rs1117602254	0.016	0.28	C	
*PVT1*	2017	296	Russian	rs2114358	0.005	2.77 (1.33–5.77)	A	29095108
*RFPL3*	2017	532	Multinational *	rs1789084	0.010	0.57	C	28569182
		532	Multinational *	rs73166319	<0.001	0.12	C	
*SLC1A4*	2017	639	Multinational *	rs759458	<0.001	3.31	G	28569182
		532	Multinational *	rs759458	0.049	1.86	G	
*TRBC*	2007	31	Belgium, Canada, The Netherlands, Italy, and the UK	rs71878	0.015	6.8 (1.45–31.9)	C	17622942
*UVRAG*	2017	639	Multinational *	rs80191572	0.002	0.20	A	28569182
		532	Multinational *	rs80191572	<0.001	0.12	A	
*ZAK*	2017	639	Multinational *	rs139890339	<0.001	0.05	C	28569182
		532	Multinational *	rs139890339	0.011	0.14	C	

* Argentina, Belgium, Bulgaria, Canada, Croatia, the Czech Republic, Estonia, Finland, France, Georgia, Germany, Hungary, Israel, Italy, Latvia, Lithuania, The Netherlands, Poland, Romania, Russia, South Africa, Spain, Ukraine, the UK, and the United States.

## Abbreviations

ALOX5AP	Arachidonate 5-lipoxygenase-activating protein gene
APC	Antigen-presenting cell
CCR5	C-C motif chemokine receptor 5 gene
CIS	Clinically isolated syndrome
CLEC16A	C-type lectin domain family 16 member A gene
CNS	Central nervous system
CTSS	Cathepsin S gene
EOMES	Eomesodermin gene
FORTE	Forty mg Efficacy of glatiramer acetate
GA	Glatiramer acetate
GALA	Glatiramer Acetate Low-frequency Administration
HLA-DRB1	Major histocompatibility complex class II DR beta-1 gene
IL1RL1	Interleukin 1 receptor-like 1 gene
IL22RA2	Interleukin 22 receptor alpha-2 gene
JNK/SAPK1	C-Jun N-terminal kinase 1/stress-activated protein kinase
MAGI2	Membrane-associated guanylate kinase WW and PDZ domains-containing 2 gene
MBP	Myelin basic protein
MS	Multiple sclerosis
NFĸΒ	Nuclear factor kappa light chain in β cells
PPMS	Primary progressive multiple sclerosis
PTP	Protein tyrosine phosphatase
PTPRT	Protein-tyrosine phosphatase receptor-type T gene
PVT1	Plasmacytoma variant translocation oncogene
RFPL3	RET finger protein-like 3 gene
RRMS	Relapsing-remitting multiple sclerosis
SLC1A4	Solute carrier family 1 member 4 gene
SPMS	Secondary progressive multiple sclerosis
Th2	T helper type 2
TRBC1	T-cell receptor beta chain constant region 1 gene
UVRAG	UV radiation resistance-associated gene
ZAK	Leucine zipper- and sterile alpha motif-containing kinase gene

## References

[1] Freal J.E., Kraft G.H., Coryell J.K. (1984). Symptomatic fatigue in multiple sclerosis. Arch. Phys. Med. Rehabil..

[2] Hauser S.L., Oksenberg J.R. (2006). The neurobiology of multiple sclerosis: Genes, inflammation, and neurodegeneration. Neuron.

[3] Krupp L.B., Alvarez L.A., LaRocca N.G., Scheinberg L.C. (1988). Fatigue in multiple sclerosis. Arch. Neurol..

[4] Lucchinetti C., Bruck W., Parisi J., Scheithauer B., Rodriguez M., Lassmann H. (2000). Heterogeneity of multiple sclerosis lesions: Implications for the pathogenesis of demyelination. Ann. Neurol..

[5] Hestvik A.L. (2010). The double-edged sword of autoimmunity: Lessons from multiple sclerosis. Toxins.

[6] Tanaka M., Vecsei L. (2020). Monitoring the Redox Status in Multiple Sclerosis. Biomedicines.

[7] Tanaka M., Toldi J., Vecsei L. (2020). Exploring the Etiological Links behind Neurodegenerative Diseases: Inflammatory Cytokines and Bioactive Kynurenines. Int. J. Mol. Sci..

[8] Arslan B., Arslan G.A., Tuncer A., Karabudak R., Dinçel A.S. (2021). Evaluation of Thiol Homeostasis in Multiple Sclerosis and Neuromyelitis Optica Spectrum Disorders. Front. Neurol..

[9] Ömerhoca S., Akkaş S.Y., İçen N.K. (2018). Multiple Sclerosis: Diagnosis and Differential Diagnosis. Arch. Neuropsychiatry.

[10] Brownlee W.J. (2019). Differential diagnosis of multiple sclerosis. Better Explan. Clin. Pract..

[11] Luca M., Chisari C.G., Zanghì A., Patti F. (2021). Early-Onset Alcohol Dependence and Multiple Sclerosis: Diagnostic Challenges. Int. J. Environ. Res. Public Health.

[12] Toscano S., Patti F. (2021). CSF biomarkers in multiple sclerosis: Beyond neuroinflammation. Neuroimmunol. Neuroinflamm..

[13] Browne P., Chandraratna D., Angood C., Tremlett H., Baker C., Taylor B.V., Thompson A.J. (2014). Atlas of Multiple Sclerosis 2013: A growing global problem with widespread inequity. Neurology.

[14] Pugliatti M., Rosati G., Carton H., Riise T., Drulovic J., Vecsei L., Milanov I. (2006). The epidemiology of multiple sclerosis in Europe. Eur. J. Neurol..

[15] Compston A., Coles A. (2008). Multiple sclerosis. Lancet.

[16] Lublin F.D., Reingold S.C., Cohen J.A., Cutter G.R., Sorensen P.S., Thompson A.J., Wolinsky J.S., Balcer L.J., Banwell B., Barkhof F. (2014). Defining the clinical course of multiple sclerosis: The 2013 revisions. Neurology.

[17] Grossman I., Avidan N., Singer C., Goldstaub D., Hayardeny L., Eyal E., Ben-Asher E., Paperna T., Pe’er I., Lancet D. (2007). Pharmacogenetics of glatiramer acetate therapy for multiple sclerosis reveals drug-response markers. Pharm. Genom..

[18] Grossman I., Knappertz V., Laifenfeld D., Ross C., Zeskind B., Kolitz S., Ladkani D., Hayardeny L., Loupe P., Laufer R. (2017). Pharmacogenomics strategies to optimize treatments for multiple sclerosis: Insights from clinical research. Prog. Neurobiol..

[19] Castro-Borrero W., Graves D., Frohman T.C., Flores A.B., Hardeman P., Logan D., Orchard M., Greenberg B., Frohman E.M. (2012). Current and emerging therapies in multiple sclerosis: A systematic review. Adv. Neurol. Disord..

[20] Huisman E., Papadimitropoulou K., Jarrett J., Bending M., Firth Z., Allen F., Adlard N. (2017). Systematic literature review and network meta-analysis in highly active relapsing-remitting multiple sclerosis and rapidly evolving severe multiple sclerosis. BMJ Open.

[21] Findling O., Sellner J. (2021). Second-generation immunotherapeutics in multiple sclerosis: Can we discard their precursors?. Drug Discov. Today.

[22] Doshi A., Chataway J. (2017). Multiple sclerosis, a treatable disease. Clin. Med..

[23] Coyle P.K. (2017). Pharmacogenetic Biomarkers to Predict Treatment Response in Multiple Sclerosis: Current and Future Perspectives. Mult. Scler. Int..

[24] Aharoni R. (2013). The mechanism of action of glatiramer acetate in multiple sclerosis and beyond. Autoimmun. Rev..

[25] Tennakoon D.K., Mehta R.S., Ortega S.B., Bhoj V., Racke M.K., Karandikar N.J. (2006). Therapeutic induction of regulatory, cytotoxic CD8+ T cells in multiple sclerosis. J. Immunol..

[26] Tsareva E., Kulakova O., Boyko A., Favorova O. (2016). Pharmacogenetics of multiple sclerosis: Personalized therapy with immunomodulatory drugs. Pharm. Genom..

[27] Montalban X., Gold R., Thompson A.J., Otero-Romero S., Amato M.P., Chandraratna D., Clanet M., Comi G., Derfuss T., Fazekas F. (2018). ECTRIMS/EAN Guideline on the pharmacological treatment of people with multiple sclerosis. Mult. Scler..

[28] Boster A.L., Ford C.C., Neudorfer O., Gilgun-Sherki Y. (2015). Glatiramer acetate: Long-term safety and efficacy in relapsing-remitting multiple sclerosis. Expert Rev. Neurother..

[29] Weinstock-Guttman B., Nair K.V., Glajch J.L., Ganguly T.C., Kantor D. (2017). Two decades of glatiramer acetate: From initial discovery to the current development of generics. J. Neurol. Sci..

[30] Ganji A., Monfared M.E., Shapoori S., Nourbakhsh P., Ghazavi A., Ghasami K., Mosayebi G. (2019). Effects of interferon and glatiramer acetate on cytokine patterns in multiple sclerosis patients. Cytokine.

[31] Johnson K.P., Brooks B.R., Cohen J.A., Ford C.C., Goldstein J., Lisak R.P., Myers L.W., Panitch H.S., Rose J.W., Schiffer R.B. (1995). Copolymer 1 reduces relapse rate and improves disability in relapsing-remitting multiple sclerosis: Results of a phase III multicenter, double-blind placebo-controlled trial. The Copolymer 1 Multiple Sclerosis Study Group. Neurology.

[32] Comi G., Filippi M., Wolinsky J.S. (2001). European/Canadian multicenter, double-blind, randomized, placebo-controlled study of the effects of glatiramer acetate on magnetic resonance imaging--measured disease activity and burden in patients with relapsing multiple sclerosis. European/Canadian Glatiramer Acetate Study Group. Ann. Neurol..

[33] Fusco C., Andreone V., Coppola G., Luongo V., Guerini F., Pace E., Florio C., Pirozzi G., Lanzillo R., Ferrante P. (2001). HLA-DRB1*1501 and response to copolymer-1 therapy in relapsing-remitting multiple sclerosis. Neurology.

[34] Bovis F., Kalincik T., Lublin F., Cutter G., Malpas C., Horakova D., Havrdova E.K., Trojano M., Prat A., Girard M. (2021). Treatment Response Score to Glatiramer Acetate or Interferon Beta-1a. Neurology.

[35] Drew L. (2016). Pharmacogenetics: The right drug for you. Nature.

[36] Zarzuelo-Romero M.J., Pérez-Ramírez C., Carrasco-Campos M.I., Sánchez-Martín A., Calleja Hernández M.A., Ramírez-Tortosa M.C., Jiménez-Morales A. (2021). Therapeutic Value of Single Nucleotide Polymorphisms on the Efficacy of New Therapies in Patients with Multiple Sclerosis. J. Pers. Med..

[37] Carrasco-Campos M.I., Pérez-Ramírez C., Macías-Cortés E., Puerta-García E., Sánchez-Pozo A., Arnal-García C., Barrero-Hernández F.J., Calleja-Hernández M., Jiménez-Morales A., Cañadas-Garre M. (2021). Pharmacogenetic Predictors of Response to Interferon Beta Therapy in Multiple Sclerosis. Mol. Neurobiol..

[38] Martínez-Aguilar L., Pérez-Ramírez C., Maldonado-Montoro M.D.M., Carrasco-Campos M.I., Membrive-Jiménez C., Martínez-Martínez F., García-Collado C., Calleja-Hernández M., Ramírez-Tortosa M.C., Jiménez-Morales A. (2020). Effect of genetic polymorphisms on therapeutic response in multiple sclerosis relapsing-remitting patients treated with interferon-beta. Mutat. Res. Rev. Mutat. Res..

[39] Carter N.J., Keating G.M. (2010). Glatiramer acetate: A review of its use in relapsing-remitting multiple sclerosis and in delaying the onset of clinically definite multiple sclerosis. Drugs.

[40] McGraw C.A., Lublin F.D. (2013). Interferon beta and glatiramer acetate therapy. Neurotherapeutics.

[41] Aharoni R. (2014). Immunomodulation neuroprotection and remyelination—The fundamental therapeutic effects of glatiramer acetate: A critical review. J. Autoimmun..

[42] From R., Eilam R., Bar-Lev D.D., Levin-Zaidman S., Tsoory M., LoPresti P., Sela M., Arnon R., Aharoni R. (2014). Oligodendrogenesis and myelinogenesis during postnatal development effect of glatiramer acetate. Glia.

[43] Hocevar K., Ristic S., Peterlin B. (2019). Pharmacogenomics of Multiple Sclerosis: A Systematic Review. Front. Neurol..

[44] Mahurkar S., Suppiah V., O’Doherty C. (2014). Pharmacogenomics of interferon beta and glatiramer acetate response: A review of the literature. Autoimmun. Rev..

[45] Comabella M., Vandenbroeck K. (2011). Pharmacogenomics and multiple sclerosis: Moving toward individualized medicine. Curr. Neurol. Neurosci. Rep..

[46] Yandava C.N., Kennedy B.P., Pillari A., Duncan A.M., Drazen J.M. (1999). Cytogenetic and radiation hybrid mapping of human arachidonate 5-lipoxygenase-activating protein (ALOX5AP) to chromosome 13q12. Genomics.

[47] Safizadeh B., Hoshyar R., Mehrpour M., Eftekhar M., Salimi V., Yazdani S., Bijari B., Khodakhah F., Tavakoli-Yaraki M. (2018). The role of expression and activity of 15-Lipoxygenase isoforms and related cytokines in patients with Multiple Sclerosis and healthy controls. J. Neuroimmunol..

[48] Achiron A., Feldman A., Gurevich M. (2009). Molecular profiling of glatiramer acetate early treatment effects in multiple sclerosis. Dis. Markers.

[49] Ross C.J., Towfic F., Shankar J., Laifenfeld D., Thoma M., Davis M., Weiner B., Kusko R., Zeskind B., Knappertz V. (2017). A pharmacogenetic signature of high response to Copaxone in late-phase clinical-trial cohorts of multiple sclerosis. Genome Med..

[50] Reeves R.H., Patch D., Sharpe A.H., Borriello F., Freeman G.J., Edelhoff S., Disteche C. (1997). The costimulatory genes Cd80 and Cd86 are linked on mouse chromosome 16 and human chromosome 3. Mamm Genome.

[51] Freeman G.J., Borriello F., Hodes R.J., Reiser H., Gribben J.G., Ng J.W., Kim J., Goldberg J.M., Hathcock K., Laszlo G. (1993). Murine B7-2, an alternative CTLA4 counter-receptor that costimulates T cell proliferation and interleukin 2 production. J. Exp. Med..

[52] Fraussen J., Claes N., Van Wijmeersch B., van Horssen J., Stinissen P., Hupperts R., Somers V. (2016). B cells of multiple sclerosis patients induce autoreactive proinflammatory T cell responses. Clin. Immunol..

[53] Hussien Y., Sanna A., Soderstrom M., Link H., Huang Y.M. (2001). Glatiramer acetate and IFN-beta act on dendritic cells in multiple sclerosis. J. Neuroimmunol..

[54] Yi C.H., Terrett J.A., Li Q.Y., Ellington K., Packham E.A., Armstrong-Buisseret L., McClure P., Slingsby T., Brook J.D. (1999). Identification, mapping, and phylogenomic analysis of four new human members of the T-box gene family: EOMES, TBX6, TBX18, and TBX19. Genomics.

[55] Parnell G.P., Gatt P.N., Krupa M., Nickles D., McKay F.C., Schibeci S.D., Batten M., Baranzini S., Henderson A., Barnett M. (2014). The autoimmune disease-associated transcription factors EOMES and TBX21 are dysregulated in multiple sclerosis and define a molecular subtype of disease. Clin. Immunol..

[56] Raveney B.J.E., Sato W., Takewaki D., Zhang C., Kanazawa T., Lin Y., Okamoto T., Araki M., Kimura Y., Sato N. (2021). Involvement of cytotoxic Eomes-expressing CD4(+) T cells in secondary progressive multiple sclerosis. Proc. Natl. Acad. Sci. USA.

[57] Kulakova O., Bashinskaya V., Kiselev I., Baulina N., Tsareva E., Nikolaev R., Kozin M., Shchur S., Favorov A., Boyko A. (2017). Pharmacogenetics of glatiramer acetate therapy for multiple sclerosis: The impact of genome-wide association studies identified disease risk loci. Pharmacogenomics.

[58] Dale M., Nicklin M.J. (1999). Interleukin-1 receptor cluster: Gene organization of IL1R2, IL1R1, IL1RL2 (IL-1Rrp2), IL1RL1 (T1/ST2), and IL18R1 (IL-1Rrp) on human chromosome 2q. Genomics.

[59] Yagami A., Orihara K., Morita H., Futamura K., Hashimoto N., Matsumoto K., Saito H., Matsuda A. (2010). IL-33 mediates inflammatory responses in human lung tissue cells. J. Immunol..

[60] Ahmadi M., Fathi F., Fouladi S., Alsahebfosul F., Manian M., Eskandari N. (2019). Serum IL-33 Level and IL-33, IL1RL1 Gene Polymorphisms in Asthma and Multiple Sclerosis Patients. Curr. Mol. Med..

[61] Carpintero R., Burger D. (2011). IFNbeta and glatiramer acetate trigger different signaling pathways to regulate the IL-1 system in multiple sclerosis. Commun. Integr. Biol..

[62] Morton S.M., Bocaccio I., Depetris D., Mattei M., Dessein A. (1997). Assignment of IL12RB2 to human chromosome 1p31.3-->p31.2 between D1S230 and D1S198. Cytogenet. Genome Res..

[63] Koch M.A., Thomas K.R., Perdue N.R., Smigiel K.S., Srivastava S., Campbell D.J. (2012). T-bet(+) Treg cells undergo abortive Th1 cell differentiation due to impaired expression of IL-12 receptor beta2. Immunity.

[64] Jana M., Mondal S., Jana A., Pahan K. (2014). Interleukin-12 (IL-12), but not IL-23, induces the expression of IL-7 in microglia and macrophages: Implications for multiple sclerosis. Immunology.

[65] Xu W., Presnell S.R., Parrish-Novak J., Kindsvogel W., Jaspers S., Chen Z., Dillon S.R., Gao Z., Gilbert T., Madden K. (2001). A soluble class II cytokine receptor, IL-22RA2, is a naturally occurring IL-22 antagonist. Proc. Natl. Acad. Sci. USA.

[66] Lindahl H., Guerreiro-Cacais A.O., Bedri S.K., Linnerbauer M., Linden M., Abdelmagid N., Tandre K., Hollins C., Irving L., Glover C. (2019). IL-22 Binding Protein Promotes the Disease Process in Multiple Sclerosis. J. Immunol..

[67] Wood J.D., Yuan J., Margolis R.L., Colomer V., Duan K., Kushi J., Kaminsky Z., Kleiderlein J.J., Sharp A.H., Ross C.A. (1998). Atrophin-1, the DRPLA gene product, interacts with two families of WW domain-containing proteins. Mol. Cell. Neurosci..

[68] Bierzynska A., Soderquest K., Dean P., Colby E., Rollason R., Jones C., Inward C.D., McCarthy H.J., Simpson M.A., Lord G.M. (2017). MAGI2 Mutations Cause Congenital Nephrotic Syndrome. J. Am. Soc. Nephrol..

[69] Wu X., Hepner K., Castelino-Prabhu S., Do D., Kaye M.B., Yuan X.J., Wood J., Ross C., Sawyers C.L., Whang Y.E. (2000). Evidence for regulation of the PTEN tumor suppressor by a membrane-localized multi-PDZ domain containing scaffold protein MAGI-2. Proc. Natl. Acad. Sci. USA.

[70] Graham M., Adams J.M. (1986). Chromosome 8 breakpoint far 3′ of the c-myc oncogene in a Burkitt’s lymphoma 2;8 variant translocation is equivalent to the murine pvt-1 locus. EMBO J..

[71] Eftekharian M.M., Ghafouri-Fard S., Soudyab M., Omrani M.D., Rahimi M., Sayad A., Komaki A., Mazdeh M., Taheri M. (2017). Expression Analysis of Long Non-coding RNAs in the Blood of Multiple Sclerosis Patients. J. Mol. Neurosci..

[72] Zeni P.F., Mraz M. (2021). LncRNAs in adaptive immunity: Role in physiological and pathological conditions. RNA Biol..

[73] Timasheva Y.R., Nasibullin T.R., Tuktarova I.A., Erdman V.V., Galiullin T.R., Zaplakhova O.V., Bakhtiiarova K.Z., Mustafina O.E. (2020). The analysis of association between multiple sclerosis and genetic markers identified in genome-wide association studies. Zhurnal Nevrol Psikhiatr Im S S Korsakova.

[74] Bonnefont J., Nikolaev S.I., Perrier A.L., Guo S., Cartier L., Sorce S., Laforge T., Aubry L., Khaitovich P., Peschanski M. (2008). Evolutionary forces shape the human RFPL1,2,3 genes toward a role in neocortex development. Am. J. Hum. Genet..

[75] Damseh N., Simonin A., Jalas C., Picoraro J.A., Shaag A., Cho M.T., Yaacov B., Neidich J., Al-Ashhab M., Juusola J. (2015). Mutations in SLC1A4, encoding the brain serine transporter, are associated with developmental delay, microcephaly and hypomyelination. J. Med. Genet..

[76] Heimer G., Marek-Yagel D., Eyal E., Barel O., Oz Levi D., Hoffmann C., Ruzzo E.K., Ganelin-Cohen E., Lancet D., Pras E. (2015). SLC1A4 mutations cause a novel disorder of intellectual disability, progressive microcephaly, spasticity and thin corpus callosum. Clin. Genet..

[77] Beall S.S., Biddison W.E., McFarlin D.E., McFarland H.F., Hood L.E. (1993). Susceptibility for multiple sclerosis is determined, in part, by inheritance of a 175-kb region of the TcR V beta chain locus and HLA class II genes. J. Neuroimmunol..

[78] Hockertz M.K., Paty D.W., Beall S.S. (1998). Susceptibility to relapsing-progressive multiple sclerosis is associated with inheritance of genes linked to the variable region of the TcR beta locus: Use of affected family-based controls. Am. J. Hum. Genet..

[79] Deussing J., Roth W., Rommerskirch W., Wiederanders B., von Figura K., Peters C. (1997). The genes of the lysosomal cysteine proteinases cathepsin B, H, L, and S map to different mouse chromosomes. Mamm. Genome.

[80] Foti Cuzzola V., Palella E., Celi D., Barresi M., Giacoppo S., Bramanti P., Marino S. (2012). Pharmacogenomic update on multiple sclerosis: A focus on actual and new therapeutic strategies. Pharm. J.

[81] Liu R., Paxton W.A., Choe S., Ceradini D., Martin S.R., Horuk R., MacDonald M.E., Stuhlmann H., Koup R.A., Landau N.R. (1996). Homozygous defect in HIV-1 coreceptor accounts for resistance of some multiply-exposed individuals to HIV-1 infection. Cell.

[82] Rottman J.B., Ganley K.P., Williams K., Wu L., Mackay C.R., Ringler D.J. (1997). Cellular localization of the chemokine receptor CCR5. Correlation to cellular targets of HIV-1 infection. Am. J. Pathol..

[83] Lynch E.A., Heijens C.A.W., Horst N.F., Center D.M., Cruikshank W.W. (2003). Cutting edge: IL-16/CD4 preferentially induces Th1 cell migration: Requirement of CCR5. J. Immunol..

[84] Nakajima H., Fukuda K., Doi Y., Sugino M., Kimura F., Hanafusa T., Ikemoto T., Shimizu A. (2004). Expression of TH1/TH2-related chemokine receptors on peripheral T cells and correlation with clinical disease activity in patients with multiple sclerosis. Eur. Neurol..

[85] Hoglund R.A., Hestvik A.L., Holmoy T., Maghazachi A.A. (2011). Expression and functional activity of chemokine receptors in glatiramer acetate-specific T cells isolated from multiple sclerosis patient receiving the drug glatiramer acetate. Hum. Immunol..

[86] Tsareva E.Y., Kulakova O.G., Boyko A.N., Shchur S.G., Lvovs D., Favorov A.V., Gusev E.I., Vandenbroeck K., Favorova O.O. (2012). Allelic combinations of immune-response genes associated with glatiramer acetate treatment response in Russian multiple sclerosis patients. Pharmacogenomics.

[87] Hakonarson H., Grant S.F., Bradfield J.P., Marchand L., Kim C.E., Glessner J.T., Grabs R., Casalunovo T., Taback S.P., Frackelton E.C. (2007). A genome-wide association study identifies KIAA0350 as a type 1 diabetes gene. Nature.

[88] Geijtenbeek T.B., Gringhuis S.I. (2009). Signalling through C-type lectin receptors: Shaping immune responses. Nat. Rev. Immunol..

[89] Rijvers L., Melief M.J., van Langelaar J., van der Vuurst de Vries R.M., Wierenga-Wolf A.F., Koetzier S.C., Priatel J.J., Jorritsma T., van Ham S.M., Hintzen R.Q. (2020). The Role of Autoimmunity-Related Gene CLEC16A in the B Cell Receptor-Mediated HLA Class II Pathway. J. Immunol..

[90] Zuvich R.L., Bush W.S., McCauley J.L., Beecham A.H., De Jager P.L., Ivinson A.J., Compston A., Hafler D.A., Hauser S.L., the International Multiple Sclerosis Genetics Consortium (2011). Interrogating the complex role of chromosome 16p13.13 in multiple sclerosis susceptibility: Independent genetic signals in the CIITA-CLEC16A-SOCS1 gene complex. Hum. Mol. Genet..

[91] Inazawa J., Itoh N., Abe T., Nagata S. (1992). Assignment of the human Fas antigen gene (Fas) to 10q24.1. Genomics.

[92] Haas J., Fritzsching B., Trubswetter P., Korporal M., Milkova L., Fritz B., Vobis D., Krammer P.H., Suri-Payer E., Wildemann B. (2007). Prevalence of newly generated naive regulatory T cells (Treg) is critical for Treg suppressive function and determines Treg dysfunction in multiple sclerosis. J. Immunol..

[93] Schwarz A., Schumacher M., Pfaff D., Schumacher K., Jarius S., Balint B., Wiendl H., Haas J., Wildemann B. (2013). Fine-tuning of regulatory T cell function: The role of calcium signals and naive regulatory T cells for regulatory T cell deficiency in multiple sclerosis. J. Immunol..

[94] Häusler D., Hajiyeva Z., Traub J.W., Zamvil S.S., Lalive P.H., Bruck W., Weber M.S. (2020). Glatiramer acetate immune modulates B-cell antigen presentation in treatment of MS. Neurol.-Neuroimmunol. Neuroinflamm..

[95] Gross E.A., Callow M.G., Waldbaum L., Thomas S., Ruggieri R. (2002). MRK, a mixed lineage kinase-related molecule that plays a role in gamma-radiation-induced cell cycle arrest. J. Biol. Chem..

[96] Liu T.C., Huang C.J., Chu Y.C., Wei C.C., Chou C.C., Chou M.Y., Chou C.K., Yang J.J. (2000). Cloning and expression of ZAK, a mixed lineage kinase-like protein containing a leucine-zipper and a sterile-alpha motif. Biochem. Biophys. Res. Commun..

[97] Hasson T., Kolitz S., Towfic F., Laifenfeld D., Bakshi S., Beriozkin O., Shacham-Abramson M., Timan B., Fowler K.D., Birnberg T. (2016). Functional effects of the antigen glatiramer acetate are complex and tightly associated with its composition. J. Neuroimmunol..

[98] Von Salome J., Gyllensten U., Bergstrom T.F. (2007). Full-length sequence analysis of the HLA-DRB1 locus suggests a recent origin of alleles. Immunogenetics.

[99] Lundberg A.S., McDevitt H.O. (1992). Evolution of major histocompatibility complex class II allelic diversity: Direct descent in mice and humans. Proc. Natl. Acad. Sci. USA.

[100] Gross R., Healy B.C., Cepok S., Chitnis T., Khoury S.J., Hemmer B., Weiner H.L., Hafler D.A., De Jager P.L. (2011). Population structure and HLA DRB1 1501 in the response of subjects with multiple sclerosis to first-line treatments. J. Neuroimmunol..

[101] Saxe D.F., Takahashi N., Hood L., Simon M.I. (1985). Localization of the human myelin basic protein gene (MBP) to region 18q22→qter by in situ hybridization. Cytogenet. Genome Res..

[102] Boggs J.M. (2006). Myelin basic protein: A multifunctional protein. Cell. Mol. Life Sci..

[103] (2012). Glatiramer Acetate. LiverTox: Clinical and Research Information on Drug-Induced Liver Injury.

[104] McAndrew P.E., Frostholm A., White R.A., Rotter A., Burghes A.H. (1998). Identification and characterization of RPTP rho, a novel RPTP mu/kappa-like receptor protein tyrosine phosphatase whose expression is restricted to the central nervous system. Mol. Brain Res..

[105] Wang Z., Shen D., Parsons D.W., Bardelli A., Sager J., Szabo S., Ptak J., Silliman N., Peters B.A., van der Heijden M.S. (2004). Mutational analysis of the tyrosine phosphatome in colorectal cancers. Science.

[106] Perelman B., Dafni N., Naiman T., Eli D., Yaakov M., Feng T.L., Sinha S., Weber G., Khodaei S., Sancar A. (1997). Molecular cloning of a novel human gene encoding a 63-kDa protein and its sublocalization within the 11q13 locus. Genomics.

[107] Afzal S., Hao Z., Itsumi M., Abouelkheer Y., Brenner D., Gao Y., Wakeham A., Hong C., Li W.Y., Sylvester J. (2015). Autophagy-independent functions of UVRAG are essential for peripheral naive T-cell homeostasis. Proc. Natl. Acad. Sci. USA.

